# The Immunometabolic Gene N-Acetylglucosamine Kinase Is Uniquely Involved in the Heritability of Multiple Sclerosis Severity

**DOI:** 10.3390/ijms25073803

**Published:** 2024-03-28

**Authors:** Serge Nataf, Marine Guillen, Laurent Pays

**Affiliations:** 1 Bank of Tissues and Cells, Hospices Civils de Lyon, Hôpital Edouard Herriot, Place d’Arsonval, F-69003 Lyon, France; 2 Stem-Cell and Brain Research Institute, 18 Avenue du Doyen Lépine, F-69500 Bron, France; 3 Lyon-Est School of Medicine, University Claude Bernard Lyon 1, 43 Bd du 11 Novembre 1918, F-69100 Villeurbanne, France

**Keywords:** multiple sclerosis, NOD2, glycolysis, monocytes, dendritic cells, NAGK, multiple sclerosis severity, multiple sclerosis heritability

## Abstract

The clinical severity of multiple sclerosis (MS), an autoimmune disorder of the central nervous system, is thought to be determined by environmental and genetic factors that have not yet been identified. In a recent genome-wide association study (GWAS), a single nucleotide polymorphism (SNP), rs10191329, has been associated with MS severity in two large independent cohorts of patients. Different approaches were followed by the authors to prioritize the genes that are transcriptionally regulated by such an SNP. It was concluded that the identified SNP regulates a group of proximal genes involved in brain resilience and cognitive abilities rather than immunity. Here, by conducting an alternative strategy for gene prioritization, we reached the opposite conclusion. According to our re-analysis, the main target of rs10191329 is N-Acetylglucosamine Kinase (NAGK), a metabolic gene recently shown to exert major immune functions via the regulation of the nucleotide-binding oligomerization domain-containing protein 2 (NOD2) pathway. To gain more insights into the immunometabolic functions of NAGK, we analyzed the currently known list of NAGK protein partners. We observed that NAGK integrates a dense network of human proteins that are involved in glucose metabolism and are highly expressed by classical monocytes. Our findings hold potentially major implications for the understanding of MS pathophysiology.

## 1. Introduction

In the last decade, a flurry of genome-wide association studies (GWAS) have allowed a fine mapping of multiple sclerosis (MS) susceptibility genes. These research efforts culminated in 2019 with a reference map of MS genetic architecture which gathered more than 200 autosomal regulatory single nucleotide polymorphisms (SNPs) associated with MS risk [1]. However, none of these SNPs were found to be associated with the severity of MS, thus leaving a fundamental question unanswered. Indeed, multiple sclerosis can manifest in a wide variety of clinical forms in terms of symptoms and prognosis. Up to 30% of patients with multiple sclerosis develop a benign form of the disease that requires little or no therapeutic intervention [2,3]. On the other side of the spectrum, aggressive forms of MS may lead to severe clinical disability in a few years. Adjusting treatments to the predicted clinical severity of MS represents thus a major challenge that genetics did not seem able to meet. In this context, the recent demonstration that a unique SNP, rs10191329, associates with MS severity [4] is undeniably a milestone discovery for at least two reasons. First of all, this finding indicates that rs10191329 could be used as a standalone genetic marker for the stratification of MS patients, whether in the context of therapeutic trials or for routine clinical management. Secondly, the association of rs10191329 with MS severity is supposed to provide major pathophysiological insights, as far as the gene(s) regulated by such an SNP is/are clearly identified. Unravelling this or these gene(s) may also deeply impact our quest for new biological markers and therapeutic targets in MS.

In their paper, Harroud et al. [4] identified two genes, namely Zinc Finger Protein 638 (*ZNF638*) and Dysferlin (*DYSF*), as the main targets of rs10191329. Importantly, such genes were prioritized firstly on the basis of proximity, i.e., the close vicinity of rs10191329 with the transcription start sites (TSS) of *ZNF638* and *DYSF*. Since both genes are highly expressed by neural cells, notably oligodendrocytes, the authors suggested then that rs10191329 impacts MS severity via the transcriptional regulation of *ZNF638* and *DYSF* in neural cells [4]. It was concluded that such a molecular mechanism may set the level of brain tissue resilience and thus clinical severity in MS patients. However, Harroud et al. [4] did not provide any experimental proof that rs10191329, depending on the allele considered, associates with distinct expression levels of *ZNF638* and/or *DYSF* in neural cells. Harroud et al. [4] further supported their “tissue resilience” hypothesis by demonstrating an inverse correlation link between the trait “years of education” and MS severity [4]. Overall, it was thus proposed that the so-called “cognitive reserve”, recognized as a protective factor in the context of Alzheimer’s disease [5,6], might also be involved in a process of brain resilience underlying the association between rs10191329 and MS severity. Such findings may immensely impact the upcoming orientation of MS therapeutic research. Actually, there is no doubt that many researchers will rely on this key paper to advocate for the development and use of neuroprotective treatments in MS.

However, we think that caution should be exercised regarding the gene prioritization strategy that was chosen by these authors. It is useful to remind that the term “gene prioritization” refers to the methods used by geneticists for determining which gene(s) is/are more likely to be transcriptionally regulated by a given SNP. This can be a complex process based on multiple parameters, including, in particular, (i) gene proximity, (ii) the relevance of candidate target genes in the context of a given pathology (at least for SNP associated with a pathological trait), (iii) the expression profile of candidate target genes in relevant cells and/or tissues and, most importantly, (iv) the measured impact of a SNP on gene expression and/or protein levels in relevant cells and/or tissues.

Here, we present gene prioritization results indicating that *ZNF638* and *DYSF* are not the main targets of rs10191329. Instead, we found that rs10191329 appears to essentially target the immunometabolic gene N-Acetylglucosamine Kinase (*NAGK*). Moreover, such an effect may operate in immune cells, not neural cells. Our data mining analyses also provide insights into the expression pattern of *NAGK* as well as the genomic and proteomic networks that associate with NAGK. We conclude that NAGK is crucially involved in the glucose metabolism of classical monocytes and that such a function likely contributes to (or interferes with) the immunoregulatory pathway linking NAGK to the innate immune receptor “nucleotide-binding oligomerization domain-containing protein 2″ (NOD2). These findings are discussed in the context of MS pathophysiology.

## 2. Results

### 2.1. The Regulatory SNP rs10191329 Targets NAGK in Immune Cells

**Figure 1:**
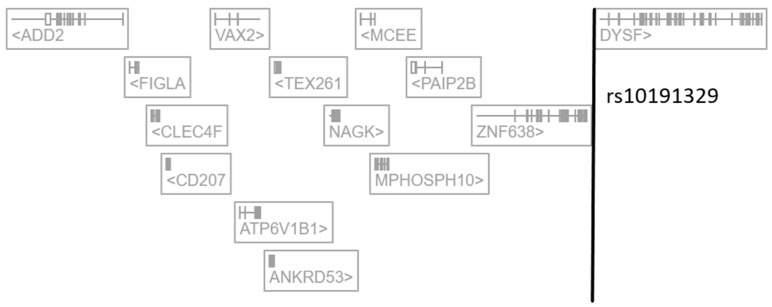
Representation of rs10191329 localization in the human genome (extracted and adapted from the Open Targets Genetics platform). *ADD2*: Adducin 2, *FIGLA*: Folliculogenesis Specific BHLH Transcription Factor, *CLEC4F*: C-Type Lectin Domain Family 4 Member F, *CD207*: CD207 Molecule, *VAX2*: Ventral Anterior Homeobox 2, *ATP6V1B1*: ATPase H+ Transporting V1 Subunit B1, *ANKRD53*: Ankyrin Repeat Domain 53, *TEX261*: Testis Expressed 261, *NAGK*: N-Acetylglucosamine Kinase, *MCEE*: Methylmalonyl-CoA Epimerase, *MPHOSPH10*: M-Phase Phosphoprotein 10, *PAIP2B*: Poly(A) Binding Protein Interacting Protein 2B, *ZNF638*: Zinc Finger Protein 638, *DYSF*: Dysferlin.

There is now a large number of expression quantitative trait loci (eQTL) studies in which links between SNPs and transcriptomics data have been explored in a multitude of cells and tissues. In this regard, the Open Targets Genetics (OTG) platform (https://genetics.opentargets.org/ (accessed on 12 February 2024)) offers the unique opportunity to simultaneously survey as many as 60 eQTL datasets generated by distinct consortia and obtained across 92 tissues and cell types [7,8]. The OTG platform is more than a catalog of previously published data as it retrospectively performs integrated statistical fine-mapping across thousands of trait-associated loci, including expression quantitative traits (eQTLs) and protein quantitative trait loci (pQTLs) [7,8]. We thus surveyed the OTG database and extracted results linking the SNP rs10191329 with expression and/or protein quantitative traits. We observed first that rs10191329 does not associate with any expression or protein quantitative trait previously described in the CNS across 18 distinct CNS regions. In contrast, rs10191329 was reported to associate with eQTLs in (i) blood cells for N-Acetylglucosamine Kinase (*NAGK*), Zinc Finger Protein 638 (*ZNF638*), Methylmalonyl-CoA Epimerase (*MCEE*), C-Type Lectin Domain Family 4 Member F (*CLEC4F*) and M-Phase Phosphoprotein 10 (*MPHOSPH10*), (ii) interferon-gamma (IFN-γ)-stimulated monocytes for *NAGK* and (iii) testis and induced pluripotent stems cells for Dysferlin (*DYSF*) (Table 1 and Figure 1). While the reported rs10191329-regulated genes actually comprise *ZNF638* and *DYSF*, it should be underscored that *NAGK* is by far the gene for which the largest size effect and most significant *p*-value are reported. Of note also, such a regulatory effect was observed in both blood cells and monocytes stimulated with interferon-gamma (IFN-γ), i.e., cell types that can be considered as relevant in the context of MS pathophysiology. Finally, according to OTG, rs10191329 associates with only one protein quantitative trait, namely the plasma levels of NAGK (beta value = −0.165, *p*-value = 2.5 × 10^−7^) (Table 1).

There is thus compelling evidence indicating that the regulatory SNP rs10191329 essentially targets *NAGK* in immune cells rather than *ZN638* or *DYSF* in neural cells.

### 2.2. Monocytes, Macrophages and Myeloid Dendritic Cells Are the Immune Cell Types Exhibiting the Highest NAGK mRNA Levels

To gain more insights into the expression pattern of *NAGK*, we surveyed the single-cell RNA-seq database provided by the Human Protein Atlas (HPA) (https://www.proteinatlas.org/) (accessed on 12 February 2024) [9]. Extracted results showed that among the six candidate genes selected above on the basis of eQTL data (Table 1), *NAGK* exhibits the highest expression levels in immune cells, notably dendritic cells, monocytes and macrophages) (Table 2). In contrast, *NAGK* is poorly expressed by neural cells, including excitatory neurons, inhibitory neurons, astrocytes, oligodendrocytes and microglia (Table 2).

Importantly, a survey of the HPA transcriptomics database dedicated to human blood cells [10] indicates that, among blood-circulating immune cells, classical monocytes (also named conventional monocytes) exhibit the highest *NAGK* mRNA levels (https://www.proteinatlas.org/ENSG00000124357-NAGK/immune+cell) (accessed on 12 February 2024). Confirming these data, the single-cell RNA-seq database run by the European Molecular Biology Laboratory (EMBL) also reports that myeloid cells, notably monocytes and dendritic cells, form the great majority of cell types to which *NAGK* was assigned by clustering analysis (Table 3).

To further assess the expression pattern of NAGK, we queried the co-expression database COEXPRESdb (https://coxpresdb.jp/) (accessed on 5 February 2024) [11] and extracted the top 100 genes exhibiting the most significant NAGK co-expression links across 25,362 samples and 1324 studies (Table S1). From this list, we explored via the enrichment platform Enrichr (https://maayanlab.cloud/Enrichr/) (accessed on 5 February 2024) [12,13] three cell type enrichment libraries that we selected on the basis of their high gene coverage (>10,000). These libraries comprise Jensen Tissues [14], Human Gene Atlas [15] and CellMarker Augmented 2021 [16]. Confirming the expression data presented above, this analysis showed that monocytes are the cell type expressing the most significant number of NAGK co-expressed genes (Table 4). In addition, from the same list of NAGK co-expressed genes, a combined survey of five libraries of pathways (Reactome [17], BioPlanet [18], KEGG [19], Panther [20] and WikiPathways [21]) showed that the phagocyte-related term “lysosome” reached the most significant enrichment (adjusted *p*-value: 0.00004).

Altogether, these data unravel the unique expression pattern of NAGK in immune cells and suggest that classical monocytes could be the main cell type affected by a rs10191329-mediated regulation of NAGK.

### 2.3. In Monocytes, NAGK Physically Interacts with a Dense Network of Proteins Involved in the Glycogenesis and Neoglucogenesis Pathway

**Figure 2:**
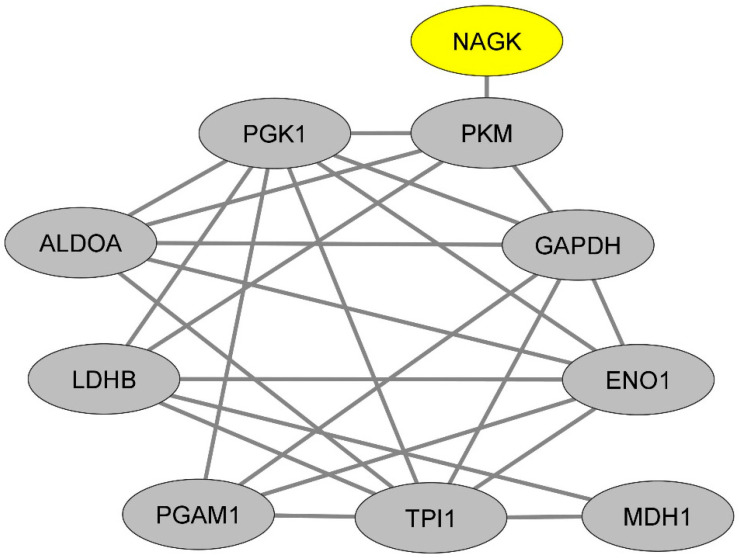
Representation of the protein–protein interaction network linking NAGK and glycolysis-related proteins.

We then sought to gain insights into the functions potentially exerted by NAGK in classical monocytes. To achieve this goal, we first queried the protein–protein interaction database BioGrid [22] to extract the currently known list of NAGK protein partners, irrespective of the cell type considered (Table S2). From this list, we then used the Enrichr platform to perform a pathway enrichment analysis based on the combined survey of five libraries of pathways (Reactome [17], BioPlanet [18], KEGG [19], Panther [20] and WikiPathways [21]). Interestingly, the most statistically significant enrichment was found for the pathway termed “glycogenesis and neoglucogenesis” (adjusted *p*-value: 0.002). This pathway comprises the following NAGK protein partners: lactate deshydrogenase A (LDHA), pyruvate kinase M1/M2 (PKM), hexokinase 2 (HK2), glutamic-oxaloacetic transaminase 1 (GOT1) and lactate dehydrogenase A like 6B (LDHAL6B). We next attempted to determine whether NAGK might interact with a network of glycolysis-related proteins in classical monocytes. To this aim, we assessed in classical monocytes the expression levels of the 45 human genes annotated with the term “glycogenesis and neoglucogenesis” according to the library of pathways “Wikipathways” [21]) (Table S3). From these 45 genes, we selected the top 20% of genes exhibiting the highest expression levels in classical monocytes (Table S4), and for each of the proteins encoded by such genes (n = 9), we extracted the currently known list of protein partners according to the database BioGrid (Tables S5–S13). Surprisingly, we found that each of these lists of protein partners were themselves highly significantly enriched in proteins involved in the “glycogenesis and neoglucogenesis” pathway (Tables S5–S13). In other words, the proteins annotated with the term “glycogenesis and neoglucogenesis” are highly connected with proteins annotated with the term “glycogenesis and neoglucogenesis”. From these experimental proteomic data, we were thus able to draw a dense network of proteins that are likely abundant in classical monocytes, are connected with NAGK and exert glycogenesis and neoglucogenesis functions (Figure 2).

A recent work showed that the main NOD2-ligand, the gut-derived postbiotic muramyl-dipeptide (MDP), needs to be phosphorylated by NAGK in order to activate NOD2 [23]. We thus thought to determine whether the MDP-mediated activation of NOD2 in classical monocytes would impact the expression levels of *NAGK* and of the nine glycolysis-related genes identified above. To this aim, we queried the NIH-run transcriptomics database Geodatasets (https://www.ncbi.nlm.nih.gov/gds) (accessed on 5 February 2024) and extracted data from the, up to now, only genomic study exploring the molecular profile of MDP-stimulated classical monocytes [24] (Ref: GSE10146). As presented in Table 5, we found that *NAGK* and eight of our nine genes of interest were significantly up-regulated following the MDP stimulation of classical monocytes. Moreover, in non-classical monocytes (also named patrolling monocytes), the impact of MDP on *NAGK* and glycolysis-related genes appeared to be either distinct (for *LDHB*, *TPI1* and *MDH1*) or less pronounced (for *NAGK*, *PKM*, *PGK1*, *ALDOA*, *PGAM1*, *ENO1* and *GAPDH*).

Altogether, these results indicate that NAGK belongs to a complex network of glycolysis-related proteins that are abundant in classical monocytes and might shape the metabolic status of classical monocytes under MDP-NOD2 activation.

### 2.4. NAGK Is Potentially Involved in the Glycosylation of Key MS-Related Innate Immune Molecules

**Figure 3:**
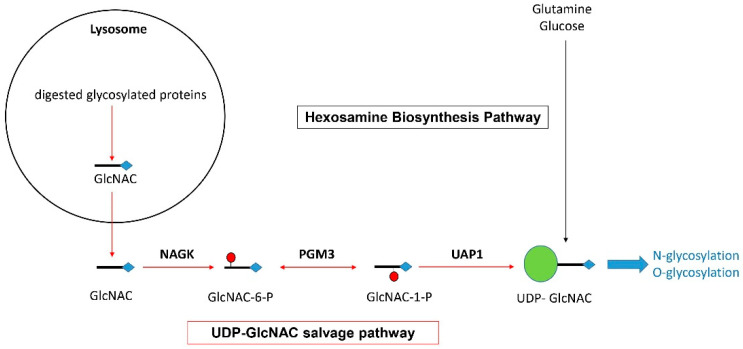
Representation of the hexosamine biosynthesis pathway and UDP-GlcNAC salvage pathway allowing the generation of UDP-GlcNAC for protein glycosylation (based on data previously published by others [25,26,27]).

One of the main functions previously assigned to NAGK is the recycling of N-acetylglucosamine (Glc-NAC) molecules generated by the lysosomal degradation of glycosylated proteins [25,26,27]. As depicted in Figure 3 and previously demonstrated by others [25,26,27], NAGK converts GlcNAC into N-acetylglucosamine 6-phosphate (GlcNAC-6-P), from which the enzymes phosphoglucomutase 3 (PGM3) and UDP-N-acetylglucosamine pyrophosphorylase (UAP1) generate Uridine diphosphate N-acetylglucosamine (UDP-GlcNAC) [25]. Since UDP-GlcNAC acts as a major glycosyl donor in a large range of glycosylation processes (including the O- and N-glycosylation of proteins), NAGK is thus involved in a crucial recycling mechanism, also called the UDP-GlcNAC salvage pathway [25,26,27]. Such a pathway supports the intracellular turnover of glycosylated proteins. In fact, it has been previously estimated that half of all amino sugars from endocytosed glycans are recycled [26]. However, UDP-GlacNAC is also generated from glucose and glutamine via the de novo hexosamine biosynthesis pathway (HBP).

In any case, it appears highly likely that NAGK participates, at least to some extent, in the process of protein glycosylation in monocytes, dendritic cells and macrophages. To obtain a general overview of such glycosylated proteins, we queried the N-GlycositeAtlas database (http://nglycositeatlas.biomarkercenter.org/) (accessed on 12 February 2024) [28]. The N-GlycositeAtlas is to our knowledge the only glycoprotein database that compiles results obtained by mass spectrometry on a large range of human cells and tissues in over 100 studies. We extracted data obtained from human blood-derived macrophages, the only immune cell type from which results are available in this database (Table S14). Interestingly, the set of 121 glycosylated proteins identified in human macrophages includes key immune molecules previously involved in MS pathophysiology. These notably comprise the complement component C3 [29,30,31], the complement C3 receptor integrin alpha-M [32], the immune checkpoint molecule “programmed cell death 1 ligand 1” (PD-L1, also named CD274) [33,34] and the human leucocyte antigen HLA-DR15 [35,36].

These results indicate that in mononuclear phagocytes, including classical monocytes, NAGK may be involved in the glycosylation of key MS-related immune molecules.

## 3. Discussion

A recent milestone paper published by Harroud et al. [4] identified the regulatory SNP rs10191329 as the only variant exhibiting a significant correlation with MS clinical severity. Confirming this finding, an independent study showed that MS patients bearing the rs10191329A allele exhibit a higher rate of brain atrophy [37]. The identification of such a unique SNP not only bears potential interest for the stratification of MS patients but also provides key insights into MS pathophysiology. However, it appears crucial to determine which gene(s) is/are transcriptionally regulated by such a SNP. In their work, Harroud et al. [4] concluded that rs10191329 regulates the expression of two candidate target genes, namely *ZNF638* and *DYSF*, which possibly shape the levels of “neurocognitive reserve” and CNS “tissue resilience” in MS patients. The arguments supporting such a conclusion may be subject to criticism. This holds particularly true with regard to the gene prioritization strategy. The main prioritization parameter chosen by the authors is indeed the spatial proximity of rs10191329A with *DYSF* and *ZNF638*. Such an argument appears relatively weak since target genes are frequently located up to 2 Mbps from their regulatory SNP [38,39,40]. In addition, based on the expression patterns of rs10191329 proximal genes, the authors infered that rs10191329 likely regulates *DYSF* and/or *ZNF638* in neural cells. Nevertheless, it is important to underscore that eQTL studies remain the gold standard for gene prioritization [41]. In this view, it is striking that across 60 eQTL datasets compiled in the OTG platform, rs10191329 does not associate with any expression quantitative traits in neural cells or tissues. According to Harroud et al. [4], the “neurocognitive reserve” hypothesis is also supported by the previous demonstration that rs10191329 associates with the trait “intelligence”. However, such an association has been found in only one study [42], reaching both a very small effect size (beta = −0.018) and a poor *p*-value (1.92 × 10^−6^) [43]. Along this line, while the authors demonstrated an inverse correlation between MS severity and the trait “years of education”, the impact of confounding factors such as lower income has not been sufficiently discussed. Indeed, as indicated in their data supplements, Harroud et al. [4] observed that, when adjusted to income, the link between the trait “years of education” and MS severity exhibited a dramatic drop in statistical significance (in cohorts 1 and 2, *p*-value = 2.09 × 10^−13^ and 4.61 × 10^−16^, respectively, without adjustment for income vs. 0.011 and 0.03 following adjustment for income). Accordingly, a recent work demonstrated that in MS patients, a lower socio-economic status defined by lower income, lower education levels and higher risk of multimorbidities, inversely correlated with an increased rate of treatment escalation [44]. It thus appears that MS severity is impacted by socio-economic status as a whole, rather than “intelligence”, “years of education” or the “neurocognitive reserve”. Finally, favoring the CNS resilience hypothesis, Harroud et al. [4] argued that homozygous carriers of the rs10191329A allele display a significantly higher number of brainstem and cortical lesions as assessed by neuropathology. Again, this observation does not formally demonstrate that rs10191329A carriers exhibit lower CNS tissue resilience. To reach such a conclusion, extended analyses would have been needed, notably, an assessment of the extent of demyelination/remyelination and axonal loss in patients harboring comparable levels of inflammation. In this regard, a recent neuroimaging study showed that rs10191329A carriers exhibit not only an increased rate of brain atrophy but also an increased volume of white matter lesions [37]. At this stage, it is thus extremely difficult to determine whether rs10191329A carriers may display a lower CNS tissue resilience or an increased immune aggressiveness of MS lesions.

According to our re-analysis, the main target of rs10191329 is not *ZNF638* or *DYSF* in neural cells but *NAGK* in monocytes. More specifically, MS patients bearing the rs10191329A allele are expected to exhibit a decreased expression of *NAGK* in immune cells, notably classical monocytes. While only poor evidence supports a role for *DYSF* and/or *ZNF638* in MS pathophysiology, several pathways, possibly intermingled, might link a decreased expression of *NAGK* to MS severity. The first one is the NAGK-MDP-NOD2 regulatory pathway. Multiple lines of evidence indeed suggest that NOD2 is an immunoregulatory receptor. Thus, inherited forms of the autoinflammatory disorder “Blau syndrome” (also named early onset sarcoidosis) are caused by NOD2 loss-of-function mutations [45,46]. Similarly, homozygous carriers of NOD2 loss-of-function mutations exhibit a >40-fold increased risk of developing the autoimmune condition Crohn’s disease [47,48,49]. Finally, in distinct experimental paradigms, including the MS murine model experimental autoimmune encephalomyelitis (EAE), the systemic administration of MDP was shown to foster immune tolerance via the activation of NOD2 in myeloid cells [50,51,52,53]. Of note, such a protective effect was found to rely, at least in part, on the MDP-induced conversion of classical monocytes into non-classical (patrolling) monocytes [24]. This point is of particular interest since classical monocytes were reported to exert pathogenic functions in EAE [54,55,56] and MS [54,57]. Overall, we propose that MS patients bearing the rs10191329A allele may experience increased clinical severity via an alteration of the NAGK-MDP-NOD2 regulatory pathway.

Our data mining results also indicate that NAGK physically interacts with a dense network of proteins involved in glucose metabolism. Interestingly, the genes encoding such proteins are highly expressed by classical monocytes and harbor increased expression levels under MDP-induced activation of NOD2 [24]. While multiple metabolic alterations have been reported in MS patients [58], our observations suggest that NAGK contributes specifically to the control of glucose metabolism in monocytes. In particular, NAGK may shape the metabolic reprogramming that accompanies the MDP-induced conversion of classical monocytes into non-classical (patrolling) monocytes.

Finally, the role of NAGK in the recycling of glycosylated proteins [25,26,27] is another potential pathway linking NAGK to MS severity. Supporting this view, the serum levels of GlcNAC, the main substrate of NAGK in the UDP-GlcNAC salvage pathway (Figure 3), were shown to inversely correlate with clinical severity in MS patients [59]. It should be emphasized, however, that low GalNAC serum levels may impact nonimmune processes, such as myelin repair [60]. Nevertheless, oral GalNAC was reported to treat EAE [61] and to exert measurable anti-inflammatory effects in MS patients [62]. In rs10191329A carriers, a decreased expression of NAGK is thus predicted to mimic the impact of low GalNAC serum levels and to induce qualitative and/or quantitative alterations of protein glycosylation in immune cells.

## 4. Materials and Methods

All bioinformatics and data mining analyses were performed at least 3 times between August 2023 and February 2024.

### 4.1. Exploration of eQTL Datasets

The Open Targets Genetics (OTG) platform was surveyed, and results linking the SNP rs10191329 with expression and/or protein quantitative traits were extracted. A total of 60 eQTL datasets generated by distinct consortia and obtained across 92 tissues and cell types were explored.

### 4.2. Mining of Transcriptomic Datasets

The NIH-run transcriptomics database Geodatasets (https://www.ncbi.nlm.nih.gov/gds) (accessed on 12 February 2024) was queried with the terms “classical monocytes” (or “conventional monocytes”) and “muramyl dipeptide”. This query returned only one study, referred to as GSE101496 [24], from which data were extracted. In other analyses, we mined the expression atlas “Human Protein Atlas” (https://www.proteinatlas.org/) (accessed on 5 February 2024), which, to our knowledge, gathers the largest sets of expression data obtained on human cells, notably via single-cell RNA-seq analyses [9]. When needed, we explored on HPA, the expression dataset dedicated to immune blood cells [10]. We also queried the single-cell expression atlas provided by the European Molecular Biology Laboratory (https://www.ebi.ac.uk/gxa/sc/home) (accessed on 5 February 2024), which compiles 147 single-cell RNA seq datasets generated from human cells. Finally, for gene co-expression analyses, we surveyed the 2023 version of the co-expression database CoXpressdb (https://coxpresdb.jp/) (accessed on 5 February 2024) [11], which provides, from any queried gene, the list of top 100 most significantly co-expressed genes as calculated across 25,362 samples from 1324 studies.

### 4.3. Mining of Proteomic Datasets

Protein–protein interactions were extracted from the Biogrid database (https://thebiogrid.org/) (accessed on 12 February 2024) [22], which hosts ~1.93 million curated protein and genetic interactions in several species. We retained only protein–protein interactions obtained via experimental approaches (i.e., not inferred from in silico analyses) and demonstrated in the human species.

### 4.4. Enrichment Analyses

Pathway enrichment analyses were performed on the web platform “Enrichr” (https://maayanlab.cloud/Enrichr/) (accessed on 5 February 2024) [12,13] by combining results obtained from 5 distinct libraries of pathways: Reactome [17], BioPlanet [18], KEGG [19], Panther [20] and WikiPathways [21]. Tissue enrichment analyses were also performed on Enrichr via the exploration of 3 cell type enrichment libraries that we selected on the basis of their high gene coverage (>10,000): Jensen Tissues [14], Human Gene Atlas [15] and CellMarker Augmented 2021 [16]. When needed, as shown in Tables S5–S13, enrichment factors were calculated, and statistical significance was assessed using the Fisher’s exact test.

## 5. Conclusions

The demonstration of an association between the SNP rs10191329 and MS severity is undeniably a milestone discovery that rightfully generates many expectations, including therapeutic ones. The transcriptional regulation of *ZNF638* and *DYSF* in neural cells was initially identified as the main mechanism linking rs10191329 with MS severity. Here, using an alternative strategy for gene prioritization, we found that rs10191329 likely targets the immunometabolic gene *NAGK* in immune cells. We provide data mining results indicating that *NAGK* may shape MS severity via three pathways: (i) the MDP-NOD2 regulatory pathway, (ii) the glycolytic pathway and (iii) the glycosylation pathway. All three pathways may essentially operate in classical monocytes, conferring to this cell type a key function in MS pathophysiology. Our findings are important and novel for at least three reasons. First, the present paper should stimulate research efforts aimed at assessing the role of monocytes in MS pathophysiology. Second, treating patients with an aggressive form of MS is still challenging nowadays and requires aggressive treatments with non-negligible side effects. Among the myriad of immune pathways that are potentially eligible for therapeutic interventions, we point to a specific mechanism, the NAGK/NOD2 pathway, identified on the basis of robust genetic findings. This should be considered as a major asset for clinical translatability. Moreover, apart from the stimulation of NOD2 via the systemic administration of NOD2 agonists, therapeutic strategies aimed at stimulating NAGK expression could be envisioned. Finally, while the development of neuroprotective treatments shows continuously increasing interest and mobilizes tremendous scientific efforts in MS research, our data indicate that the genetic inheritability of MS severity may not be linked to CNS tissue protection and/or repair. On the contrary, to date, the only SNP which associates with MS severity appears to essentially set the intensity of neuroinflammation, not tissue resilience.

## Figures and Tables

**Table 1:** Results from expression quantitative trait loci (eQTL) studies reported for rs10191329 in the Open Targets Genetics (OTG) platform.

Tissue or Cell Type	Regulated Gene	Distance from TSS	Beta Value	*p*-Value
blood cells	*NAGK*	385,525 bp	−0.316	6.7 × 10^−101^
	*ZNF638*	173,308 bp	+0.166	3.2 × 10^−51^
	*MCEE*	319,630 bp	−0.123	3.6 × 10^−34^
	*CLEC4F*	>500 Kb	+0.0573	1.4 × 10^−8^
	*MPHOSPH10*	319,559 bp	−0.0511	4.2 × 10^−7^
IFN-γ-stimulated monocytes	*NAGK*	385,525 bp	−0.0972	1.9 × 10^−6^
testis	*DYSF*	3692 bp	−0.290	2.3 × 10^−6^
induced pluripotent stem cells	*DYSF*	3692 bp	−0.263	5.0 × 10^−9^

TSS: transcription start site.

**Table 2:** Expression levels of *NAGK*, *ZNF638*, *MCEE*, *CLEC4F*, *MPHOSPH10* and *DYSF* as assessed by single-cell RNA-seq analysis of human immune or neural cells according to the Human Protein Atlas.

Gene (Gene Symbol and HPA URL)	Mean Normalized TPM in Immune Cells	Mean Normalized TPM in Neural Cells
*NAGK* https://www.proteinatlas.org/ENSG00000124357-NAGK/single+cell+type (accessed on 12 February 2024)	dendritic cells: 119.6 monocytes: 85.4 macrophages: 104.9 T-cells: 25.4 B-cells: 44.2	excitatory neurons: 11.1 inhibitory neurons: 14.5 astrocytes: 14.2 microglia: 25 oligodendrocytes: 22.3
*ZNF638* https://www.proteinatlas.org/ENSG00000075292-ZNF638/single+cell+type (accessed on 12 February 2024)	dendritic cells: 48.7 monocytes: 53.8 macrophages: 52.5 T-cells: 60.4 B-cells: 61.9	excitatory neurons: 277.2 inhibitory neurons: 257 astrocytes: 216.1 microglia: 215.6 oligodendrocytes: 576.6
*MCEE* https://www.proteinatlas.org/ENSG00000124370-MCEE/single+cell+type (accessed on 12 February 2024)	dendritic cells: 12.1 monocytes: 5.9 macrophages: 10.1 T-cells: 9.7 B-cells: 7.8	excitatory neurons: 12.8 inhibitory neurons: 13.9 astrocytes: 13 microglia: 13.4 oligodendrocytes: 19.4
*CLEC4F* https://www.proteinatlas.org/ENSG00000152672-CLEC4F/single+cell+type (accessed on 12 February 2024)	dendritic cells: 0 monocytes: 13.9 macrophages: 3.6 T-cells: 0.1 B-cells: 0	excitatory neurons: 0.2 inhibitory neurons: 0.1 astrocytes: 0 microglia: 0 oligodendrocytes: 0.1
*MPHOSPH10* https://www.proteinatlas.org/ENSG00000124383-MPHOSPH10/single+cell+type (accessed on 12 February 2024)	dendritic cells: 42 monocytes: 27.8 macrophages: 21.1 T-cells: 43.6 B-cells: 35.2	excitatory neurons: 9.5 inhibitory neurons: 10.9 astrocytes: 7.7 microglia: 14.3 oligodendrocytes: 14.8
*DYSF* https://www.proteinatlas.org/ENSG00000135636-DYSF/single+cell+type (accessed on 12 February 2024)	dendritic cells: 0.4 monocytes: 3.5 macrophages: 10 T-cells: 0.7 B-cells: 1.3	excitatory neurons: 33.6 inhibitory neurons: 12.5 astrocytes: 0.9 microglia: 16.1 oligodendrocytes: 58.4

TPM: transcripts per kilobase million.

**Table 3:** Occurrence of *NAGK* as a gene marker in single-cell RNA-seq studies according to the European Molecular Biology Laboratory (EMBL) single-cell RNA-seq database.

PMID	Assigned Cell Types
27864467	Blood pre-conventional dendritic cells
29808007	Monocytes and dendritic cells
31597962	Fetal liver hematopoietic cells
30692988	Tonsils dendritic cells
30402542	Decidual macrophages and dendritic cells
31594933	Blood monocytes
29352091	Effector memory CD4+ T lymphocytes
33352111	Neonatal liver monocytes
32351704	Blood, liver or spleen dendritic cells
30737144	Skin regulatory T-cells

PMID: PubMed Identifier.

**Table 4:** Results from cell and tissue enrichment analyses performed on the list of top 100 *NAGK* co-expressed genes.

Library	Cell Type	Adjusted *p*-Value
Jensen Tissues	Monocyte	6.205 × 10^−17^
	Intestine	3.604 × 10^−14^
	Natural killer cell	8.235 × 10^−14^
	Blood	2.823 × 10^−13^
	Blood platelet	5.428 × 10^−13^
Human Gene Atlas	CD33+ myeloid cell	0.001403
	CD56+ natural killer cell	0.007790
	CD14+ monocytes	0.009458
	Whole blood	0.01149
CellMarker Augmented 2021	Pancreatic Islet cell	1.508 × 10^−7^
	Basal cell	0.0004272
	Monocyte from feta kidney	0.0008851
	Liver regulatory T-cell	0.0008851

**Table 5:** Fold changes of mRNA levels obtained by RNA-seq analysis of MDP-stimulated vs. control cells in classical and non-classical monocytes.

Gene	Classical Monocytes	Non-Classical Monocytes
*NAGK*	1.53	1.28
*PKM*	1.53	1.33
*PGK1*	1.86	1.66
*ALDOA*	1.70	1.33
*LDHB*	−0.8	NS
*PGAM1*	1.6	1.5
*TPI1*	NS	1.25
*MDH1*	1.5	NS
*ENO1*	1.68	1.58
*GAPDH*	2.4	1.65

Data were extracted from the Geodataset GSE10146 [24]. NS: not statistically significant.

## Data Availability

All the data explored in this article are freely available via the distinct databases which are precisely described in the Section 4.

## Supplementary Materials

The supporting information can be downloaded at https://www.mdpi.com/article/10.3390/ijms25073803/s1.
